# A Comparison of Survival and Secondary Contraction in Expanded Versus Conventional Full-Thickness Skin Grafts: An Experimental Study in Rats

**Published:** 2012-04-24

**Authors:** As'adi Kamran, Fatemi Mohammad Javad, Fazeli Sahram, Mousavi Seyed Jaber

**Affiliations:** ^a^Burn Research Center, Tehran University of Medical Sciences, Tehran, Iran; ^b^Kermanshah University of Medical Sciences, Kermanshah, Iran; ^c^Tehran University of Medical Sciences, Tehran, Iran

## Abstract

**Aim:** The aim of this experimental study is based on a comparison between the effect of expanded versus nonexpanded full-thickness skin grafts (FTSGs) on survival and secondary wound contraction. **Methods:** A total of 20 quadrangular-shaped, full-thickness skin defects with dimensions of 2 × 3 cm were created on the backs of 10 rats. Two groups were specified in this study depending on the coverage of the aforementioned defect areas using expanded versus conventional full-thickness grafts (n = 10 per group). The survival area of each graft and the amount of graft/wound contraction (secondary contraction) were measured by computerized planimetry on days 0, 7, 30, and 90. **Results:** The mean graft areas achieved were 5.8 ± 0.6, 4.2 ± 1.5, and 1.2 ± 1.1 cm^2^ (*P* < .001) in the expanded FTSGs group and 5.3 ± 0.9, 3.3 ± 1.3, and 0.98 ± 0.8 cm^2^ (*P* < .001) in the nonexpanded FTSGs group on days 7, 30, and 90, respectively. Graft area reductions (secondary contraction) were 1.7 ± 0.9 and 2.1 ± 1.2 cm^2^ (*P* = .8) on day 30 and 2.1 ± 1.3 and 2.86 ± 0.8 cm^2^ (*P* = .3) on day 90 in the expanded and nonexpanded groups, respectively. **Conclusion:** According to this study, FTSGs that were harvested from tissue expansion revealed biological behaviors that were comparable with those of conventional grafts.

Full-thickness skin grafts (FTSGs) have been demonstrated to minimize wound contraction in multiple species.^1^^-^3 This process of secondary contraction is the consequence of the biological behavior of the graft and wound bed. For this reason, FTSGs are utilized for the reconstruction of defects where primary closure, healing by secondary intention, or healing with a split-thickness skin graft would produce a functionally or aesthetically unfavorable result^4^; however, anytime that the donor area of an FTSG cannot be closed or adequate skin of a similar color and texture to that undergoing expansion is not available, expansion of the donor site FTSG harvesting can be considered.^5^^-^8 Although the clinical application of tissue expansion typically involves the utilization of local flaps, myocutaneous, island, and free flaps have also been utilized.^9^ The resurfacing of certain anatomical regions (upper and lower eyelids, nose, upper and lower lips, chin, and hands) requires delicate coverage without obscuring the underlying fine contours and the preservation of function. In such circumstances, FTSGs are a primary reconstructive modality^5^^-^7^,^^10^ and provide durable and aesthetic results.^8^ The expansion of the donor site and the harvesting of the expanded FTSG may be a useful reconstructive measure in the following settings: (1) if there is a limited area in the vicinity of the defect to place an expander, (2) the area to be resurfaced may be too ample to enable primary closure of the donor site, and (3) the flap coverage is not sufficient for the associated reconstructive requirements due to bulkiness or a high complication rate.

There are few experimental studies concerning the biological behavior of expanded FTSGs in the literature. This study presents an animal model to compare survival area and secondary contraction between expanded and nonexpanded FTSGs.

## MATERIALS AND METHODS

In this experimental study, a total of 10 Sprague-Dawley male rats that each weighed between 350 and 400 g were used. All of the selected animals were the same age and were disease free. All experimental interventions and animal care were performed in accordance with the Institutional Animal Care and Use Committee of Tehran University of Medical Sciences.

**Figure 1:**
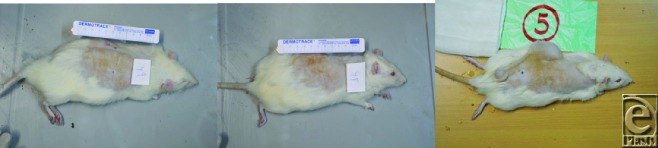
Tissue expansion placement.

**Figure 2:**
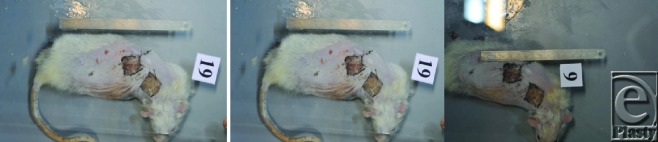
After skin grafts.

Each rat was initially anesthetized with an intramuscular injection of ketamine hydrochloride at 90 mg/kg (10% ketamine, Alfasan Lab, Worden, Holland) and xylazine at 9 mg/kg (2% xylazine, Alfasan lab, Woerden, Holland), followed by a single prophylactic dose of cephazoline (60 mg/kg). Prior to wounding, the dorsal surface of each rat was shaved with clippers and aseptically prepared with ethanol and a povidone-iodine solution, followed by an evaluation of anesthesia depth using the pinch flexion/withdrawal test. A rectangle-type, 25-cc tissue expander (Silimed, Rio de Janiro, Brazil) was inserted through a 20-mm, full-thickness horizontally oriented incision, wherein a subcutaneous pocket that was correlated with the dimensions of the expander was created immediately external to the (deep) muscle fascia (Fig 1). The tiny port of the expander was placed around the caudal end of the vertebral column over the bony prominence, such that fluid could be effortlessly injected and easily withdrawn. The incision was closed with interrupted 4-0 nylon sutures. After a lag period of 5 days, the tissue expander was inflated via the injection of 5 cc of sterile normal saline every 4 days over a period of 7 weeks. The mean total volume was around 60 cc. For approximately 2 weeks, the tissue remained fully expanded without further injection. After complying with the previous preoperative measures, second-stage surgery was performed. Initially, a 2 × 3-cm skin graft was outlined over the hemispheric, domed, and expanded flap. The demarcated area was simultaneously removed using the tissue expander. The donor site was closed using nylon 4-0 sutures. Two 2 × 3-cm, quadrangular areas of full-thickness skin were harvested from 2 distinct areas of the dorsal surface (Fig 2). For one of the donor defects, the harvested, nonexpanded skin graft was directly applied to the original site. The FTSG that had been isolated from expanded tissue was used to cover the other defect on the dorsal surface of the animal. The skin grafts of both areas were immobilized with tie-over dressings. After an interval of 7 days, the dressings were changed. Digital photographs were taken once the skin grafts had been positioned (prior to the application of the tie-over dressings) and also when they were uncovered 7 days later. All photographs were taken with a Nikon D300 camera and a 60-mm Nikon Macro Lens with a magnification of 1:10 and by the same independent photographer. All digital photographs were calibrated by a scale in each picture, which was based on the same distance between the subject and the camera (80 cm). The 2 important parameters of this experimental study include (1) graft area, which is the total surface area of the FTSGs (expanded vs nonexpanded); and (2) the zone of necrosis, which is the graft loss surface area that was analyzed via an image analysis system that used ImageJ software, V.1.40 (NIH). Following calibration, ImageJ created a value for the demarcated area on each digital photograph (the graft area and the zone of necrosis) that was converted to an area in square centimeters. The aforementioned indices were determined on the basis of square centimeters. The degree of “Take” of each group of FTSGs was determined by a single independent observer that used the same image-analysis system (ImageJ). After 1 month and 3 months, the degrees of graft/wound contraction were similarly measured by the same observer and analysis system (ImageJ).

### Statistical analysis

A paired-sample *t* test and an independent-samples *t* test were used to compare parametric variables. Data are presented as the mean ± SD. A *P* < .05 was considered to be significant. All statistical analyses were performed using SPSS 16.0 (Statistical Package for Social Sciences) for Microsoft Windows.

## RESULTS

Initially, 22 FTSGs were photographed in 11 rats. During the course of the study, one rat died from unknown causes. All of the remaining 20 grafts in 10 rats were analyzed in 2 distinct expanded versus nonexpanded groups. The mean weight gain of the rats at the end of the first month was 5%. There were no differences between the 2 groups in terms of skin texture, hair growth, color match, or evenness with the surrounding skin (*P* = .13). The survival areas of the grafts in the 2 groups were observed after 1 week (Table 1), and there was no significant difference in the percent area of graft loss between the expanded and nonexpanded groups (P = 0.103). We found that the average graft/wound contraction did not differ among the 3 different periods of time (7, 30, and 90 days) in either FTSG group (*P* > .05). The final mean wound size reached 1.2 cm^2^ in the expanded FTSG group and 0.9 cm^2^ in the nonexpanded group. The area of the necrosis zone (after a graft dressing change, eg, 7 days) was 0.2 cm^2^ in the expanded animals and 0.7 cm^2^ in the nonexpanded animals; the difference in the graft loss areas between the 2 groups was significant (*P* = .04). Overall, no statistically significant differences in the clinical behavior of the expanded or conventional full-thickness grafting techniques were observed after 30 or 90 days (Table 2).

## DISCUSSION

This experimental study comparatively investigated the effect of tissue expansion on FTSG viability and secondary contraction. According to this study, expanded FTSG exhibited survival areas and secondary contractions that were comparable with those of nonexpanded FTSGs.

Secondary contraction and graft take are the most significant determinants of FTSG outcome.^11^ By respecting the inherent tendency of rat skin to rapidly contract with minimal scar formation,^12^^-^14 we developed a valuable model that explains graft/wound contraction. Although expanded FTSGs have been used in clinical practice, we found a limited number of related studies in the literature.^5^^-^8 Furthermore, the outcome of expanded FTSGs has not been previously analyzed. For this reason, an experimental study was designed to evaluate the secondary contraction and the “take” of expanded FTSGs. In addition, a comparative review with conventional FTSGs was performed. According to our study, the patterns of “take” and survival area of both expanded and conventional FTSGs appear to be identical. The viabilities of acute and conventionally expanded FTSGs have displayed the same results in a previously investigated animal model.^10^ Overall, structural skin alterations as a result of tissue expansion do not adversely affect graft take. This may be explained by the causative role of “serum imbibition” in graft survival. Furthermore, skin that is derived from tissue expansion appeared healthy despite the presence of microscopic and biomechanical features that differ from those of nonexpanded skin.^15^ According to the results of our study, expanded and nonexpanded FTSGs reveal comparable degrees of secondary contraction. It has been proven that dermis thickness and the relative graft thickness together play a determining role in the secondary contraction of FTSGs; however, by considering the rapid, widespread thinning of the dermis during skin expansion, similar patterns of secondary contraction were detected in expanded and conventional FTSGs in our study. This contrary result may be explained in accordance with the original hypothesis that secondary contraction primarily results from wound bed contraction instead of contraction of the graft itself.^11^ In this study, expanded and nonexpanded grafts were applied over the back of the same rat. Consequently, the interaction of identical graft beds with 2 different types of FTSGs led to similar observations of secondary contraction; however, it is noticeable that the process of healing of each graft/wound inevitably affects those of other wounds.^5^ The significant merits of these graft/wound contraction experimental studies must be applied in the clinic.^11^ Furthermore, to minimize the deleterious effects of secondary graft contraction, accurate determination of the areas and relative thicknesses of the graft and dermis will be necessary.

## CONCLUSION

In this experimental study, we have demonstrated the effect of tissue expansion on the outcomes of expanded FTSGs versus conventional FTSGs. No difference in graft survival area was detected between the expanded and nonexpanded FTSGs. We have shown that expanded FTSGs exhibited the same degree of secondary contraction in comparison with that of conventional FTSGs. For this reason, the liberal use of expanded FTSGs may be considered when primary closure of the donor site is not possible or when adequate skin of similar color and texture to that undergoing expansion is not available.

## Figures and Tables

**Table 1:** A comparison of graft areas in the expansion and nonexpansion groups

Time of evaluation	Expansion group, (mean ± SD)	Nonexpansion group, (mean ± SD)	*P*
Baseline of the study, cm^2^	6	6	…
First week, cm^2^	6.01 ± 1.01	5.3 ± 0.9	.12
First month, cm^2^	3.3 ± 1.4	4.3 ± 1.6	.16
Third month, cm^2^	0.98 ± 1.4	1.2 ± 1.1	.68

**Table 2:** The comparison of wound contraction in 2 groups

Time of evaluation	Expansion group, (mean ± SD)	Nonexpansion group, (mean ± SD)	*P*
First month, cm^2^	1.75 ± 0.9	2.1 ± 1.3	.8
Third month, cm^2^	2.13 ± 1.3	2.86 ± 0.8	.3
